# The Ability to Generate Senescent Progeny as a Mechanism Underlying Breast Cancer Cell Heterogeneity

**DOI:** 10.1371/journal.pone.0011288

**Published:** 2010-06-24

**Authors:** Mine Mumcuoglu, Sevgi Bagislar, Haluk Yuzugullu, Hani Alotaibi, Serif Senturk, Pelin Telkoparan, Bala Gur-Dedeoglu, Burcu Cingoz, Betul Bozkurt, Uygar H. Tazebay, Isik G. Yulug, K. Can Akcali, Mehmet Ozturk

**Affiliations:** 1 BilGen Genetics and Biotechnology Center, Department of Molecular Biology and Genetics, Bilkent University, Ankara, Turkey; 2 INSERM - Université Joseph Fourrier, CRI U823, Grenoble, France; 3 Department of Surgery, Ankara Numune Research and Teaching Hospital, Ankara, Turkey; Health Canada, Canada

## Abstract

**Background:**

Breast cancer is a remarkably heterogeneous disease. Luminal, basal-like, “normal-like”, and ERBB2+ subgroups were identified and were shown to have different prognoses. The mechanisms underlying this heterogeneity are poorly understood. In our study, we explored the role of cellular differentiation and senescence as a potential cause of heterogeneity.

**Methodology/Principal Findings:**

A panel of breast cancer cell lines, isogenic clones, and breast tumors were used. Based on their ability to generate senescent progeny under low-density clonogenic conditions, we classified breast cancer cell lines as senescent cell progenitor (SCP) and immortal cell progenitor (ICP) subtypes. All SCP cell lines expressed estrogen receptor (ER). Loss of ER expression combined with the accumulation of p21^Cip1^ correlated with senescence in these cell lines. p21^Cip1^ knockdown, estrogen-mediated ER activation or ectopic ER overexpression protected cells against senescence. In contrast, tamoxifen triggered a robust senescence response. As ER expression has been linked to luminal differentiation, we compared the differentiation status of SCP and ICP cell lines using stem/progenitor, luminal, and myoepithelial markers. The SCP cells produced CD24+ or ER+ luminal-like and ASMA+ myoepithelial-like progeny, in addition to CD44+ stem/progenitor-like cells. In contrast, ICP cell lines acted as differentiation-defective stem/progenitor cells. Some ICP cell lines generated only CD44+/CD24-/ER-/ASMA- progenitor/stem-like cells, and others also produced CD24+/ER- luminal-like, but not ASMA+ myoepithelial-like cells. Furthermore, gene expression profiles clustered SCP cell lines with luminal A and “normal-like” tumors, and ICP cell lines with luminal B and basal-like tumors. The ICP cells displayed higher tumorigenicity in immunodeficient mice.

**Conclusions/Significance:**

Luminal A and “normal-like” breast cancer cell lines were able to generate luminal-like and myoepithelial-like progeny undergoing senescence arrest. In contrast, luminal B/basal-like cell lines acted as stem/progenitor cells with defective differentiation capacities. Our findings suggest that the malignancy of breast tumors is directly correlated with stem/progenitor phenotypes and poor differentiation potential.

## Introduction

Human breast tumors are heterogeneous, both in their pathology and in their molecular profiles. Gene expression analyses classify breast tumors into distinct subtypes, such as luminal A, luminal B, ERBB2-positive (ERBB2+) and basal-like [1], [2], [3]. The prognosis and therapeutic response of each subtype is different. Luminal A cancers are mostly estrogen receptor-α-positive (ER+) and sensitive to anti-estrogen therapy, with the best metastasis-free and overall survival rates. Luminal B tumors have an incomplete anti-estrogen response and lower survival rates. Basal-like and ERBB2+ tumors are ER- and display the worst survival rates [2], [3]. The patterns of genetic changes such as chromosomal aberrations and gene mutations observed in breast tumors indicate that breast tumorigenesis does not follow a stepwise linear progression from well-differentiated to poorly differentiated tumors with cumulative genetic aberrations [4]. This suggests that different breast tumor subtypes do not represent different stages of tumor progression, but rather represent the cells from which they initiate [4]. The mammary gland is composed of differentiated luminal and myoepithelial cells that are generated from multi-lineage, luminal-restricted, and myoepithelial-restricted progenitors originating from a hypothetical breast epithelial stem cell. Thus, different types of breast cancers might originate from such stem or progenitor cells at a given stage of commitment and differentiation, as observed in hematological malignancies [4], [5]. Without compromising the author's hypothesis, it is also possible that the molecular heterogeneity of breast cancer is due to subtle differences in the ability of tumor-initiating cells to generate differentiated progeny.

Epithelial cells isolated from mammary gland cells undergo two successive senescence states in cell culture, termed “stasis” and “agonescence” [6], [7]. In contrast to normal mammary epithelial cells, established breast cancer cells are immortal by definition. They may owe this phenotype of immortalization to genetic and epigenetic inactivation of senescence checkpoints and reactivation of telomerase reverse transcriptase expression [7]. Either in relation to these changes or independently, breast cancer cells may also present a stem/progenitor phenotype that is less subjected or resistant to senescence barriers. However, the abundance of non-tumorigenic and differentiated cells both in breast tumors and cell lines strongly suggests that replicative immortality cannot be assigned to all cells within a tumor or a cancer cell line, and that spontaneous senescence after a limited number of population doublings (PD) is likely to occur. If this hypothesis is correct, then the rate of generation of senescent progeny may reflect the potential of a cancer stem/progenitor cell to produce terminally differentiated progeny. We tested this hypothesis using a panel of luminal and basal-like breast cancer cell lines (Table S1). Although a single cell line is not representative of breast tumor heterogeneity, a panel of cell lines might reproduce the heterogeneity that is observed in primary breast tumors, albeit with some limitations [5], [8]. Therefore, we hoped that in vitro studies with a panel of cell lines might help to better understand breast tumor heterogeneity.

Our senescence tests allowed us to classify breast cancer cell lines as senescent cell progenitor (SCP) and immortal cell progenitor (ICP) subtypes. We also show that senescent progeny are observed exclusively in ER-positive cells, as a result of ER inactivation, partly mediated with p21^Cip1^ protein. The ability to produce senescent progeny was associated with the ability to produce luminal-like and myoepithelial-like progeny from stem/progenitor-like cells. In contrast, most of the cell lines lacking senescent progeny were also unable to generate differentiated progeny. Finally, we show that SCP-subtype cells cluster with luminal A and “normal-like” breast tumor types and are less tumorigenic, whereas ICP-subtype cell lines cluster with luminal B and basal-like tumor types and are more tumorigenic.

## Materials and Methods

### Ethics Statement

We used archival tumor samples remaining from a previous study by BB and IGY described in Gur-Dedeoglu et al. [9], for which the use of the tissue material was approved by the Research Ethics Committee of Ankara Numune Research and Teaching Hospital (decision date: 04/07/2007). The tumor samples in this study were used anonymously. All animals received care according to the Guide for the Care and Use of Laboratory Animals. All animal experiments have been pre-approved by the Bilkent University Animal Ethics Committee (Decision No: 2006/1; Decision date: 10/5/2006).

### Clinical samples and cell lines

Freshly frozen tumor specimens were collected at Ankara Numune Hospital. Breast cancer cell lines used in this study were obtained from ATCC (http://www.atcc.org) and listed in Table S1. Cell line authenticity was verified by short tandem repeat profiling, as recommended by ATCC (Dataset S1). Isogenic clones from the T47D (n = 20) cell line were obtained from single cell-derived colonies. Briefly, cells were plated in 96-well plates to obtain single colonies in fewer than 70% of the wells. Isolated colonies were then transferred to progressively larger wells, and to T25 flasks. Clones were subcultivated weekly at 1∶4 dilution ratios, and maintained in culture for 25–30 passages to reach >60 PD before testing.

### Primary antibodies

The following antibodies were used: anti-CD44 (559046; BD Pharmingen), anti-CD24 (sc53660; Santa Cruz,), anti-ASMA (ab7817; Abcam), anti-CK19 (sc6278; Santa Cruz,), anti-p21^Cip1^ (OP64; Calbiochem), anti-p16^Ink4a^ (NA29, Calbiochem), anti-ERα (sc8002; Santa Cruz).

### Low-density clonogenic assays

Cells were seeded as low-density on coverslips in six-well plates (500–2000 cells, according to plating efficiency) and allowed to grow in DMEM supplemented with 10% fetal calf serum (FCS), with medium change every three days, until they formed colonies of a few hundred cells. Depending on the cell line, this took one to two weeks. For bromodeoxyuridine (BrdU) incorporation assays, cells were labeled for 24 h prior to immunocytochemistry, as described previously [10].

### Immunocytochemistry

For simple immunoperoxidase assays, cells were fixed with cold methanol for five minutes, then blocked with 10% FCS in phosphate-buffered saline (PBS) for 1 hour. This was followed by incubation with a primary antibody for 1 h. Cells were then washed with PBS three times and subjected to immunostaining using the Dako-Envision-dual-link system and the liquid diaminobenzidine (DAB) substrate chromogen system (Dako, CA, USA), according to the manufacturer's instructions. Hematoxylin was used as a counter-stain when the visualization of cells was necessary. For SABG-immunoperoxidase co-staining studies, unfixed cells were first subjected to SABG assay, and then fixed prior to immunostaining assays. Hematoxylin counter-staining was omitted for co-staining experiments, unless cells were negative for SABG staining.

### Immunoblot analyses

Cell pellets were incubated in an NP-40 lysis buffer containing 50 mM Tris–HCl, pH 8.0, 250 mM NaCl, 0.1% Nonidet P-40, and a protease inhibitor cocktail (Roche) for 30 minutes in a cold room. Cell lysates were then cleared by centrifugation, and a Bradford assay was performed to quantify their protein concentration. 30 µg of protein was denatured and resolved by SDS-PAGE using 10% or 12% gels. The proteins were then transferred to the PVDF or nitrocellulose membranes. Membranes were treated for 1 h with a blocking solution of TRIS-buffered saline containing 0.1% Tween-20 and 5% non-fat milk powder (TBS-T) and probed with a primary antibody for 1 h. Next, membranes were washed three times with TBS-T and incubated with an HRP-conjugated secondary antibody for 1 h. Immunocomplexes were then detected by an ECL-plus (Amersham) kit on the membrane. α-tubulin was used as an internal control.

### SABG assay and BrdU/SABG co-staining

SABG activity was detected as described [11], except that cells were counterstained with eosin or nuclear fast red following SABG staining. For BrdU/SABG co-staining, cells were first labeled with BrdU (10 µg/ml) for 24 h in a freshly added culture medium as described [10]. Next, cells were subjected to a SABG assay, fixed in 70% methanol, and subjected to BrdU immunostaining.

### Estrogen and tamoxifen treatment

Cells were seeded under low-density clonogenic conditions onto coverslips in six-well plates, and cultivated in a standard culture medium for seven to eight days. Then, cells were fed with phenol red-free DMEM (Gibco) supplemented with 5% charcoal-stripped FCS for 48 h, followed by two successive 48 h treatments with 10^−9^ M estrogen (E2; 17β-estradiol; Sigma), 10^−6^ to 10^−9^ M 4-hydroxytamoxifen (4OHT; Sigma) or an ethanol vehicle, under the same conditions. Colonies were then subjected to a SABG assay. Each experimental condition was conducted in triplicate and experiments were repeated three times.

### Generation of estrogen receptor-overexpressing clones

T47D-iso23 cells were transfected with the expression vector pCMV- ERα [12] or an empty vector, using FuGENE-6 (Roche). ER overexpressing and control clones were selected with 500 µg/ml G418 for three weeks. Isolated single cell-derived colonies were picked and expanded in the presence of G418.

### Lentiviral infection and generation of p21^Cip1^ knockdown clones

We used mission shRNA plasmid pLKO.1<-puro-p21 (NM_000389.2-640s1c1, Sigma) for p21^cip1^ knockdown experiments. The Control vector shRNA-pGIPz-SCR-puro and a helper packaging mix (Invitrogen) were also used. HEK293T was co-transfected with the appropriate vector and packaging mix, using the CalPhos Mammalian Transfection Kit (Clontech) and following the manufacturer's instructions. After 48 h of culture, virus-containing culture media were collected, filtered, and used to infect T47D-iso23 cells. After 4 h of infection, stable cells were selected with 1 µg/ml puromycin for seven days.

### *Nude* mice tumorigenicity and in vivo senescence assays

T47D and MDA-MB-231 cells (5×10^6^) were injected subcutaneously into CD-1 *nude* mice (Charles River). Females (n = 5 for each cell line) and males (n = 4 for each cell line) were used. Tumor sizes were measured up to 47 days post-injection. In addition, four tumors from each cell line were analyzed for the presence of senescent cells by SABG staining, as described previously [10].

### Cluster analysis

The two-channel microarray data containing 8102 cDNA genes/clones generated by Sorlie et al. [2] were downloaded from the Stanford Microarray Database (SMD) (http://genome-www.stanford.edu/MicroArray/). In the downloading process, the “log (base 2) of R/G Normalized Ratio (median)” parameter was used for data filtering. We have median-centered expression values for each array. We selected arrays and genes with greater than 75% good data (representing the amount of data passing the spot criteria). Sixty-eight tissue samples were obtained according to this criterion and annotated with the subtypes described by the authors, found in the “Supplementary Information” of the data set in SMD. The expression values of “500 gene signature,” defined by the authors, were extracted from the data. Gene expression profiles of 31 breast cancer cell lines performed by Charafe-Jauffret et al. [13], using the whole-genome cDNA microarray Affymetrix HGU-133 plus 2, was obtained from the “Supplementary Table” of the article. The authors filtered genes with low and poorly measured expression, and with low expression variation, retaining 15, 293 genes. After log transformation of the data, we median normalized the data arrays in R language, using the Bioconductor biostatistical package (www.r-project.org/ and www.bioconductor.org/). The “500 gene signature” tumor data [2] and the normalized breast-cancer cell line data [13] were combined with respect to probe IDs using a set of customized perl routines (source codes are available upon request). A set of 175 genes was common. “Median center” normalization of genes was done for the merged data set for the total samples. We performed unsupervised hierarchical clustering with the 99 samples (the 31 breast cell line [13] and 68 breast tumor [2] samples) by the pair-wise complete-linkage hierarchical clustering parameter, using the Gene-Pattern program. The Pearson correlation method was used for distance measurements. Clustering was visualized by java treeview, again using Gene-Pattern (http://www.broad.mit.edu/cancer/software/genepattern/).

### Statistical analyses

Significant differences were evaluated using unpaired Student's t test for compared samples sizes of 10 or higher. Otherwise, one-tailed Fisher's exact test was used with 2×2 tables; *P<*0.05 was considered statistically significant. On the graphical representation of the data, y-axis error bars indicate the standard deviation for each point on the graph.

## Results

### Classification of breast cancer cell lines as senescent-cell progenitor and immortal-cell progenitor subtypes

**Figure 1:**
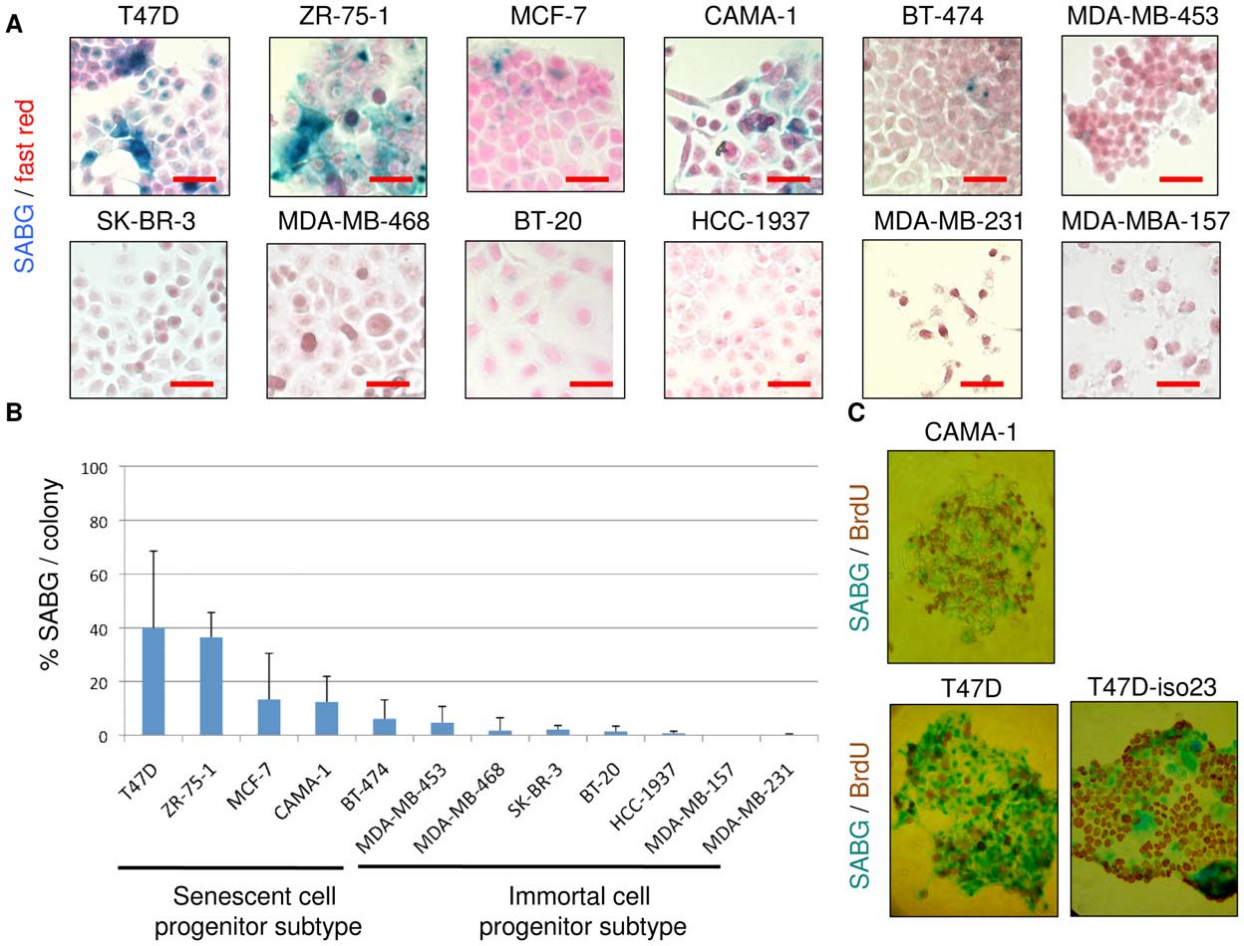
Classification of breast cancer cell lines as senescent cell progenitor and immortal cell progenitor subtypes. (**A**) Examples of SABG staining for senescence of breast cancer cell line colonies obtained after plating at low-density clonogenic conditions. Breast cancer cell lines (Table S1) were plated to obtain a few hundred colonies with 1–2 weeks of cell culturing and were subjected to SABG assay, followed by counterstaining with nuclear fast red. T47D, ZR-75-1, MCF-7, CAMA-1 and BT-474 generated heterogeneous colonies composed of with SABG+ and SABG- cells (shown here), but also fully negative and/or fully positive colonies. All other cell lines produced only SABG- colonies (<5% SABG+ cells). Scale bar: 50 µm. (**B**) Classification of breast cancer cell lines as senescent cell progenitor (SCP) and immortal cell progenitor (ICP) subtypes by quantification of the ability to generate senescent progeny. Cell lines with a mean of SABG+ cells higher than 5% were termed SCP, and the other cell lines as ICP. Colonies that were generated and stained as described in (**A**) were counted manually to calculate % SABG+ cells. At least 10 colonies were counted for each cell line. Error bars represent mean ± SD. (**C**) SABG+ senescent cells displayed terminal growth arrest. CAMA-1, T47D and T47D-iso23 colonies were generated as described in (**A**), labeled with BrdU for 24 h in the presence of freshly added culture medium, and subjected to SABG/BrdU double-staining. SABG+ cells are BrdU-, and *vice versa*. T47D-iso23 is a clone derived from T47D. Note that parental T47D and T47D-iso23 clones displayed similar staining features.

Clonogenic assays have been successfully used to test the generation and self-renewal abilities of phenotypically distinct progeny of mammary stem/progenitor cells [14]. We previously applied this technique to test the ability of cancer cells to produce progeny with replication-dependent senescence arrest [10]. Cells were plated under low-density clonogenic conditions and cultivated for one to two weeks until individual cells performed eight to ten PDs and generated isolated colonies composed of several hundred cells. This method permits tracing progeny generated by a few hundred cells under the same experimental conditions. We explored a panel of 12 breast cancer cell lines, composed of luminal (n = 7) and basal (n = 5) subtypes (Table S1). Cell lines formed two groups, according to the presence of senescent cells in isolated colonies. One group of cell lines generated colonies with high rates of senescence, while others did not produce appreciable amounts of senescent cells. Representative pictures of colonies subjected to the SABG assay are shown in Fig. 1A. The percent of SABG+ progeny was calculated by manual counting of at least 10 different colonies for each cell line. Colonies derived from five cell lines generated SABG+ cells at high rates (means: 5-40%) Senescence rates were negligible (means <5%) in the progeny of the remaining seven cell lines. The first group, the senescent cell progenitor subtype, included T47D, BT-474, ZR-75-1, MCF-7, and CAMA-1 cell lines. The second group, the immortal cell progenitor subtype, included MDA-MB-453, BT-20, SK-BR-3, MDA-MB-468, HCC1937, MDA-MB-231 and MDA-MB-157 (Fig. 1B).

In order to verify whether the occurrence of senescent cells in the SCP group was intrinsic to each cell line or due to the presence of a side population, we generated clones from the T47D (n = 20) cell line, and subjected them to the SABG assay at different intervals. All clones acted similarly to the parental T47D cell line with similar rates of SABG+ progeny. No clone gained the ICP phenotype. More importantly, none of the clones tested over a long period of time (>60 PDs) entered full senescence (data not shown), unlike normal mammary epithelial cells that undergo two stages of senescence arrest over a period of ∼20 PDs [7]. The SABG assay can provide false-positive responses, especially when cells remain under confluence for a long period [15]. Although all our tests used low-density clonogenic conditions, we wanted to confirm the senescence arrest by a long-term (24 h) BrdU labeling assay under mitogenic conditions, as senescent cells in permanent cell cycle arrest cannot incorporate BrdU under these conditions [16]. Co-staining of cells for SABG and BrdU from CAMA-1, T47D, and T47D-iso23 colonies provided clear indication that the great majority of SABG+ senescent cells were BrdU-, whereas non-senescent BrdU+ cells were usually SABG- (Fig. 1C). These findings indicated that SABG+ senescent cells were at the terminal differentiation stage with an irreversible loss of DNA synthesis ability. Our observations also indicated that SABG and BrdU tests could be used alternatively to identify senescent (SABG+/BrdU-) and immortal (SABG-/BrdU+) cells under our experimental conditions.

### Senescent cell progenitor phenotype association with p21^Cip1^ expression

**Figure 2:**
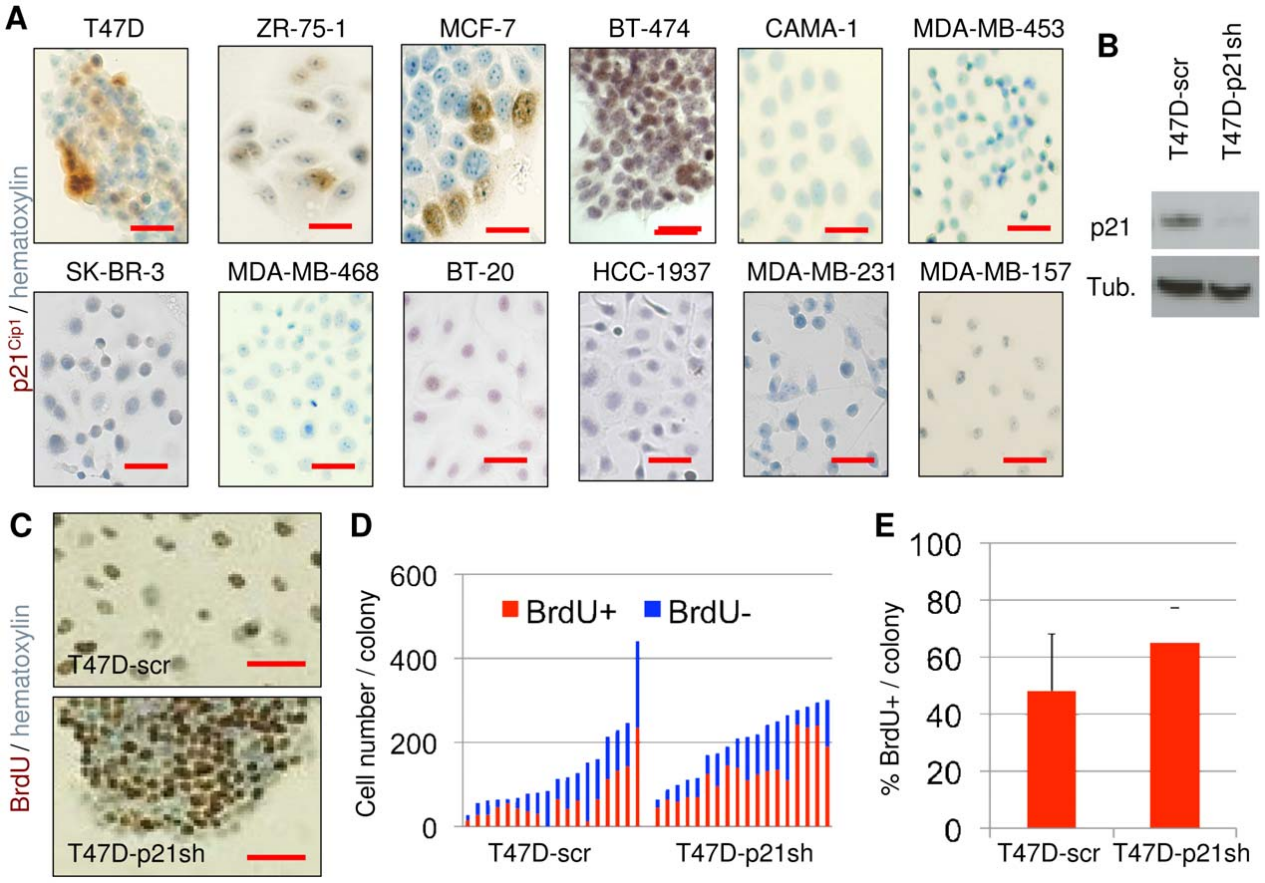
Growth arrest observed in senescent cell progenitors was inhibited by p21^Cip1^ silencing. (**A**) Four of five senescent SCP cell lines (top four from left) generated colonies with heterogeneous expression of p21^Cip1^; in contrast none of seven ICP cell lines generated p21^Cip1^ cells. Colonies were immunostained for p21^Cip1^, with hematoxylin used as counterstain. Scale bar: 50 µm. (**B–E**) p21^Cip1^ silencing inhibited the production of the terminally arrested progeny of SCP cells. (**B**) shRNA-mediated inhibition of p21^Cip1^ expression. T47D cells were infected with lentiviral vectors encoding p21^Cip1^ shRNA or scrambled shRNA to generate T47D-p21sh and T47D-scr stable cell lines, and tested for p21^Cip1^ knockdown by western blotting. (**C–E**) The ability to generate growth-arrested cells was inhibited by p21^Cip1^ knockdown. Colonies were generated from respective cell lines, labeled with BrdU for 24 h, immunostained for BrdU, and slightly counterstained with hematoxylin to visualize BrdU+ and negative cells. Scale bar: 50 µm. (**C**). Individual colonies were manually counted for quantification of % BrdU cells. Each bar represents one colony (**D**). The silencing of p21^Cip1^ caused a significant increase (*P = *0.0043) in % ratios of BrdU+ cells (**E**). Mean % BrdU+ cells (± SD) values were calculated from data presented in (**D**). Error bars represent mean ± SD. Tub.; α-tubulin.

p16^Ink4a^ and p21^Cip1^ (in a p53-dependent manner or independently) have been shown to be mediators of senescence arrest in different cells, including mammary epithelial cells [6], [15], [17], [18], [19]. We therefore analyzed the expression of p16^Ink4a^ and p21^Cip1^ in the cell line panel. Heterogeneously positive nuclear p21^Cip1^ immunoreactivity was observed in four of the five SCP cell lines, but not in any of the seven ICP cell lines (Fig. 2A). The association of p21^Cip1^ expression with the SCP subtype was statistically significant (*P* = 0.01). We also compared the expression of p16^Ink4a^. Three of five SCP cell lines displayed heterogeneously positive immunostaining, whereas three of seven ICP cell lines displayed homogenously positive staining (Fig. S1). The difference of p16^Ink4a^ expression between the two groups was not significant (*P* = 1). These observations indicated that the SCP phenotype was associated with p21^Cip^ expression in breast cancer cell lines.

To test whether p21^Cip1^ was directly involved in the senescence observed in SCP cells, we first performed p21^Cip1^/SABG staining in T47D-iso23 cells (hereafter termed T47D). p21^Cip1^, but not p16^Ink4a^ staining, was associated with SABG staining (Fig. S2). Next, we generated two derivative cell lines following infection of T47D with lentiviral vectors encoding p21^Cip1^ shRNA (T47D-p21sh) or a scrambled control (T47D-scr). Following the demonstration of p21^Cip1^ knockdown in T47D-p21sh cells by western blot assay (Fig. 2B), both cell lines were plated under low-density plating conditions, colonies were grown for 10 days, and subjected to SABG and BrdU staining. It was not possible to quantify SABG+ cells in T47D-p21sh cells because they formed tight clusters in culture (data not shown). We therefore used BrdU staining as an alternative method for senescent cell quantification (Fig. 2C). Randomly selected colonies were counted for the number of BrdU+ and BrdU- cells (Fig. 2D). The T47D-scr cell line generated BrdU+ progeny at a rate of 48±20% per colony (n = 18). Under the same conditions, T47D-p21sh cells displayed BrdU+ progeny at a rate of 65±12% per colony (n = 18), with a significant (*P = *0.0043) increase in the number of cells escaping terminal arrest (Fig. 2E). These results indicated that p21^Cip1^ was responsible, at least partly, for inducing the senescence observed in the progeny of T47D cells.

### The control of senescent cell progeny generation by an estrogen receptor

**Figure 3:**
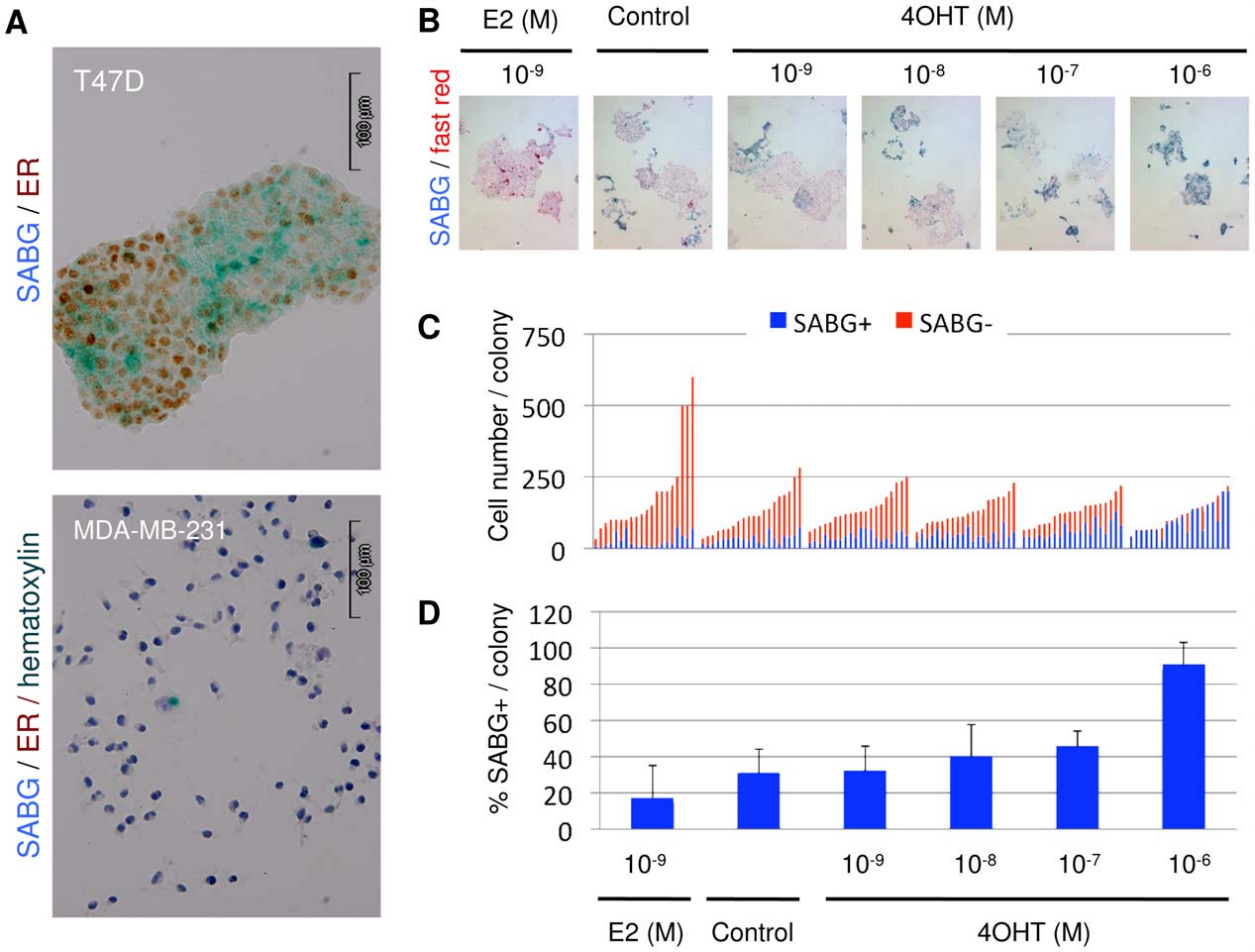
Generation of senescent cell progeny was controlled by the estrogen receptor-α. (**A**) SCP cells (T47D) expressed nuclear ER. Colonies were co-stained for senescence by SABG and for ER expression by immunoperoxidase. The MDA-MB-231 cell line was used as a negative control. (**B–D**) The production of senescent progeny in SCP cells was inhibited by estrogen (E2), but enhanced by tamoxifen (4OHT) treatment. After plating in low-density clonogenic conditions, T47D cells were grown in standard cell culture medium for seven days, followed by phenol-free DMEM complemented with charcoal-treated fetal calf serum for two days, and then cultivated for four days in the presence of E2, OHT, or an ethanol vehicle (control). Colonies were subjected to SABG staining (**B**). Total and SABG+ and SABG- cells were counted from 20 randomly selected colonies (**C**), and mean % SABG+ cells (± SD) values were calculated (**D**). Error bars represent mean ± SD. The inhibition of senescence by E2 and its activation by OHT was statistically significant when compared to ethanol-complemented control cells (*P* values 0.0093, 0.0002 and <0.0001 for 10^−9^ M E2, 10^−7^ M OHT and 10^−6^ M OHT, respectively).

As stated above, p21^Cip1^ is a downstream target of p53 for senescence, but T47D cells do not express wild-type p53 (Table S1). Estrogen inhibits p21^Cip1^ expression [20] by c-Myc-mediated repression [21], *MYC* gene being a direct target of ER complex [22]. We therefore tested whether ER could be involved in the senescence observed in T47D cells. The data shown in Fig. 3A indicates that T47D cells displayed nuclear ER immunoreactivity in their great majority, but some progeny was ER-. More interestingly, these ER- cells tended to be SABG+, suggesting that senescence occurred in T47D cells as a result of ER loss. Next, we tested whether experimentally modifying ER activity in T47D cells had any effect on senescence response. After plating at low-density clonogenic conditions, cells were grown in a regular cell culture medium that contained weakly estrogenic phenol red [23] for seven days in order to obtain visible colonies. The culture medium was then changed with phenol-free DMEM complemented with charcoal-treated FCS, grown for two more days, and then cultivated for four more days in the presence of E2 (10^−9^ M), OHT (10^−9^ M to 10^−6^ M), or an ethanol vehicle as control. Colonies were subjected to SABG staining (Fig. 3B). Total and SABG+ cells were counted from 20 randomly selected colonies for each treatment (Fig. 3C). Colonies grown in a phenol-free charcoal-treated control medium complemented with an ethanol vehicle only displayed 31±13% SABG+ cells. Complementing this medium with 10^−9^ M E2 generated colonies with 17±18% SABG+ cells. Senescence inhibition by E2 was nearly 50% and statistically significant when compared to the ethanol-complemented control cells (*P = *0.0093). In contrast to E2, OHT provoked a dose-dependent increase in the proportion of SABG+ cells. At the maximum dose used (10^−6^ M OHT), 90±13% of colony-forming cells displayed a SABG+ signal (Fig. 3D), indicating that tamoxifen-mediated inactivation of ER can induce almost a complete senescence response in these cells (*P<*0.0001). The increase in senescence rate was also significant with 10^−7^ M OHT (*P = *0.0002).

**Figure 4:**
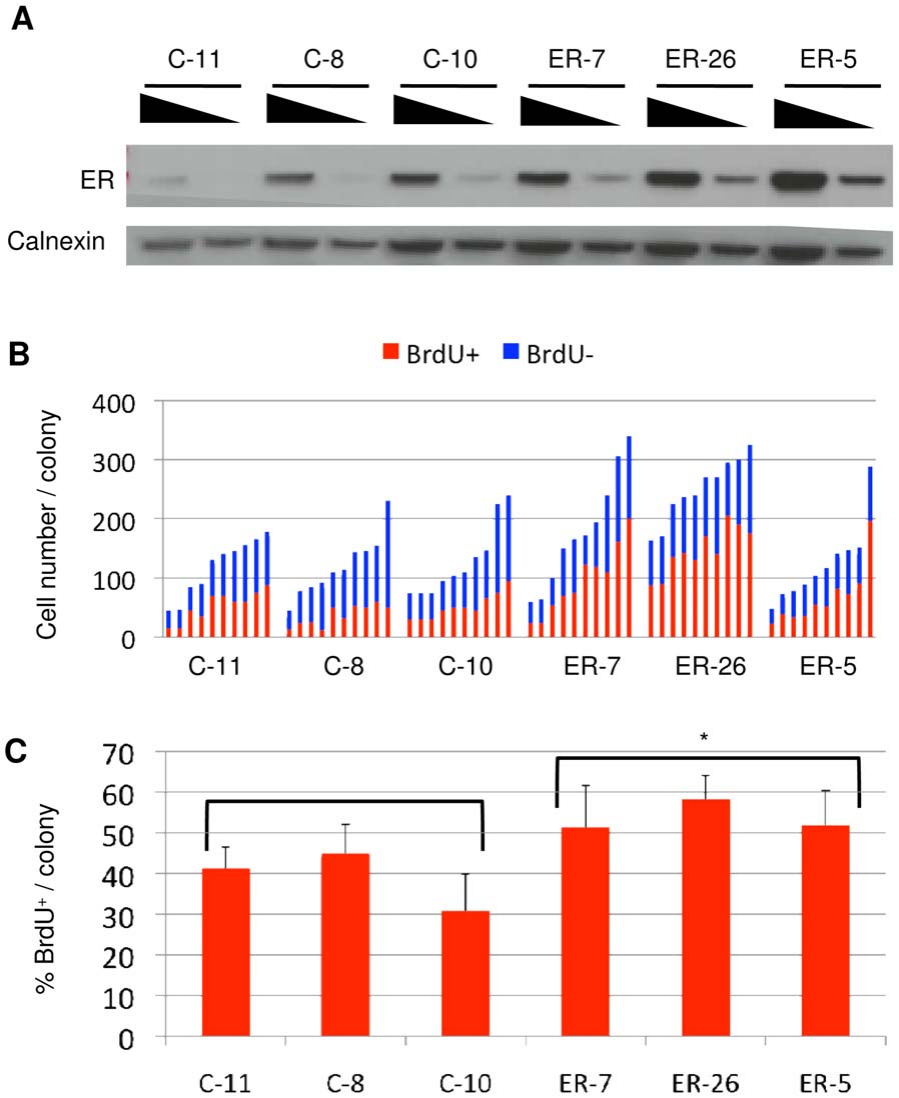
Overexpression of estrogen receptor-α inhibited the production of terminally arrested progeny. (**A**) ER-overexpressing (ER-5, ER-7, ER-26) and control (C-8, C-10, C-11) clones were established from T47D cells and tested for ER expression by western blotting using decreasing amounts of total proteins. Calnexin was used as loading control. (**B–C**). Colonies were generated, labeled with BrdU for 24 h and immunostained for BrdU (shown in Fig. S3). Individual colonies were manually counted for quantification of % BrdU cells (**B**), and mean % BrdU+ cells values were calculated (**C**). Error bars represent mean ± SD. ER overexpression caused a significant increase in % ratio of BrdU+ cells (*three ER clones *versus* three controls; *P = *0.034).

Our findings strongly suggested that the senescence observed in the SCP T47D cell line was due to a loss of expression and/or function of ER in a subpopulation of the progeny of these cells. For confirmation, we constructed ER-overexpressing stable clones from T47D cells. The thhree clones with the highest ER expression were selected. In addition, three clones with endogenous expressions of ER were selected from stable clones obtained with an empty vector (Fig. 4A). Progeny obtained from these six clones were tested by BrdU assay (Fig. S3). Randomly selected colonies (n = 10) from each clone were evaluated for total and BrdU+ number of cells (Fig. 4B). Consistently higher levels of BrdU+ cells were observed with clones ectopically expressing the ER protein (Fig. 4C). Overexpression of ER resulted in a significant increase in the BrdU+ progeny (*P = *0.034). The protective effect of ER overexpression was not as important as the senescence-promoting effects of ER inhibition. This was not unexpected, since the parental cells used for the ER overexpression studies were already expressing high levels of endogenous ER (Fig. 4A), displaying a baseline anti-senescence activity due to the serum estrogen and phenol red found in the cell culture medium.

The close relationship between ER and senescence in the ER+ T47D cell line, and the highly effective treatment of ER+ breast tumors with tamoxifen, which induced senescence in our experimental model, suggested that senescence induction might be a relevant mechanism involved in anti-estrogen treatments. As fresh tumor tissues cannot be obtained from tamoxifen-treated patients for obvious ethical reasons, we analyzed untreated ER+ breast tumor samples for evidence of spontaneously occurring in vivo senescence. We screened a panel of 12 snap-frozen ER+ breast tumor tissues from 11 patients for senescence by an SABG assay. The mean age of the patients was 58±12 yrs, with a mixed menopause status (Table S2). Two tumors (17%) displayed SABG+ cells that were scattered within the tumor area (Fig. S4). Thus, ER+ breast tumors also produced senescent progeny in vivo, but at a lower rate.

### Senescent cell progenitor and immortal cell progenitor subtypes' abilities to differentiate into luminal and myoepithelial cell types

The cellular specificity of ER expression in the mammary epithelial cell hierarchy is poorly understood. Previous data suggests that normal ER+ cells may represent either relatively differentiated luminal cells with limited progenitor capacity or primitive progenitors with stem cell properties in the luminal cell compartment [4], [24], [25]. Based on the close association between senescence (which can be considered a manifestation of terminal differentiation) and loss of ER positivity, we hypothesized that ER+ SCP cells may differ from ER- ICP cells by their differentiation potential. We surveyed a few hundred single-cell-derived colonies from each of the 12 cell lines for production of stem/progenitor-like, luminal-like, and myoepithelial-like cells. We used CD44 as a positive stem/progenitor cell marker [26], [27], CD24, ER, and CK19 as luminal lineage markers [27], [28], [29], and ASMA as a myoepithelial lineage marker [29].

**Figure 5:**
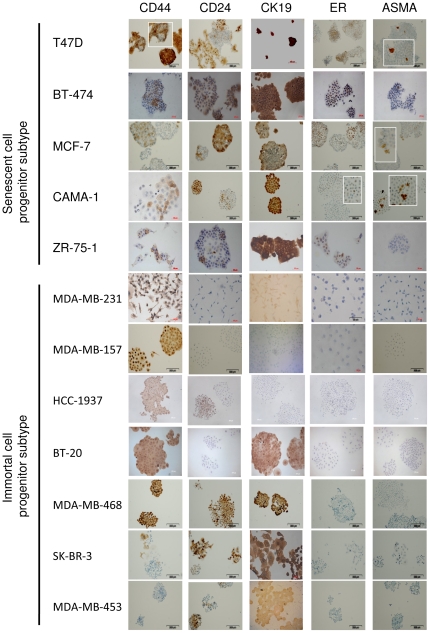
Senescent cell progenitor and immortal cell progenitor subtypes greatly differed in their ability to differentiate into luminal and myoepithelial lineage cells. Senescent cell progenitor and immortal cell progenitor subtype cell lines were studied by immunoperoxidase staining using CD44 and markers for luminal epithelial (CD24, CK19, ER) and myoepithelial (ASMA) lineages. Insets: magnified views of positive cells. Both subtypes have CD44+ cells. Senescent cell progenitor cell lines produced both progenitor-like (CD44+; CD24−), as well as ER+ luminal-like and ASMA+ myoepithelial-like cells (except ZR-75-1 for myoepithelial-like cells). Immortal cell progenitor cell lines were defective for generation of ER+ luminal-like or ASMA+ myoepithelial-like cells. Moreover, five of seven cell lines could not generate CD44- cells. The expression of CD24 and CK19 markers did not differ significantly between the two subgroups, except that some immortal cell progenitor subtype cell lines did not express CD24 or CK19.

Representative examples of marker studies by immunoperoxidase staining in SCP and ICP cell lines are shown in Fig. 5. All five SCP cell lines displayed a heterogeneous pattern of positivity for CD44; some colonies were fully positive, some fully negative, and others were composed of both positive and negative cells. CD44/CD24 double immunofluorescence studies with the T47D cell line indicated that SCP cells produce also CD44+/CD24- stem/progenitor cells, as expected (data not shown). In sharp contrast, five of the seven ICP cell lines generated only fully positive CD44 colonies, indicating they do not produce CD44- cells. One cell line was totally CD44-. Only one cell line displayed a pattern similar to that of SCP cell lines. A comparison of the two subtypes indicated that the ability to generate both CD44+ and CD44- progeny was significantly associated with the SCP phenotype (*P = *0.0046). All five SCP cell lines displayed heterogeneous, but mostly positive ER immunostaining, whereas all seven ICP cell lines never generated ER+ cells. The expression of ER was also significantly associated with the SCP subtype (*P = *0.0012), as well as the ability to produce ASMA+ progeny (*P = *0.0046). The ICP cell lines did not generate ASMA+ cells, while four out of five SCP cell lines generated rare ASMA+ cells under low-density clonogenic conditions. Interestingly, the abundance of ASMA+ cells was much higher in the two SCP cell lines that were tested at high cell density (Fig. S5). This suggests that either the production of ASMA+ cells is enhanced at high cell density, or these myoepithelial-like cells display limited survival under long-term culture conditions. We did not find a strong association between the expression of CD24 and CK19 markers and cell subtype. All five SCP cell lines and three ICP cell lines generated heterogeneously staining colonies for CD24 expression. Similarly, all five SCP cell lines, as well as three ICP cell lines, expressed CK19, but homogenously.

### Typical features of senescent progenitor and immortal progenitor breast cancer cell lines

**Figure 6:**
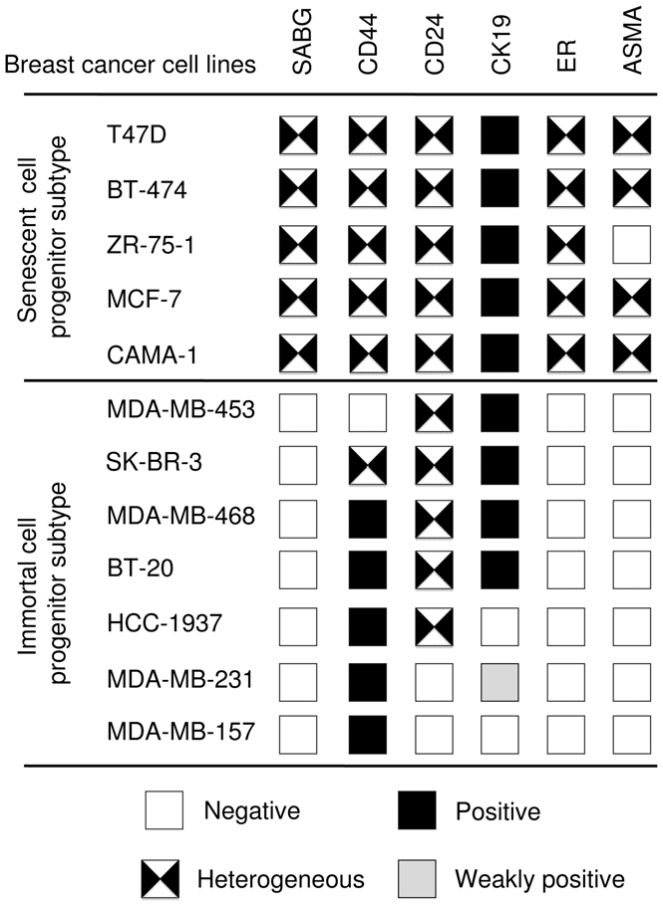
Typical features of senescent progenitor and immortal progenitor breast cancer cell lines.

As summarized in Fig. 6, SCP and ICP subtype cell lines displayed several subtype-specific features. All SCP cell lines produced differentiated and senescent cells, in addition to putative CD44+/CD24− stem/progenitor cells. Indeed, all of them produced ER+ and CD24+ luminal-like cells and most of them (n = 4/5) also produced ASMA+ myoepithelial-like cells. In contrast, five of the seven ICP cell lines never produced CD44- cells, suggesting they cannot generate differentiated progeny under the experimental conditions tested. In confirmation of this hypothesis, four ICP cell lines only produced CD44+/CD24−/ER-/CK19-/ASMA- stem/progenitor-like, but never differentiated cells. Furthermore, all seven ICP cell lines were unable to produce ASMA+ myoepithelial-like, ER+ luminal-like or SABG+ senescent cells. CD24 and CD19 luminal lineage markers were expressed in three cell lines, one of which was fully positive for the CD44 stem/progenitor marker.

### SCP and ICP subtype cell lines correlate with distinct breast tumor subtypes

**Figure 7:**
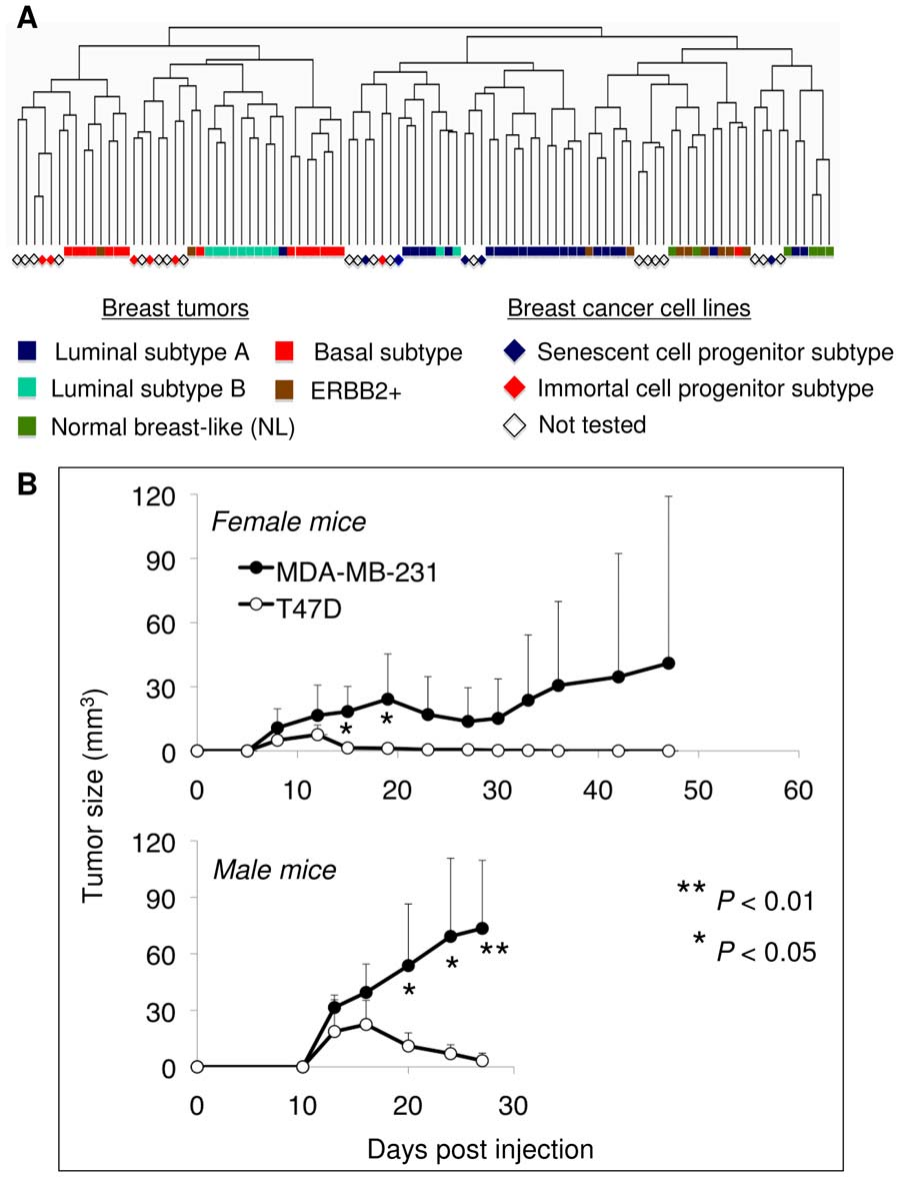
Senescence cell progenitor and immortal cell progenitor subtype cell lines correlate with distinct breast tumor subtypes. (**A**) Unsupervised hierarchical clustering of breast tumor and cell line gene expression data were obtained from Sorlie et al [2] and Charafe-Jauffret et al [25]. Dendrogram displaying the relative organization of tumor and cell line data demonstrated that ICP cell lines cluster with basal and luminal A tumors in the same branch, except for MDA-MB-453. In contrast, SCP cell lines clustered with luminal A (BT-474, CAMA-1, MCF-7, T47D) and “normal-like” tumors (ZR-75-1). No data was available for MDA-MB-468. A dendrogram with sample IDs was provided in Fig. S6. The “intrinsic gene set” data generated by Sorlie et al. [2] was used to filter cell line data generated by Charafe-Jauffret et al. [13]. A set of 175 genes was common between the two data sets. Sixty-eight tumors and 31 cell lines were subjected to pair-wise complete-linkage hierarchical clustering and distance measurements. (**B**) Immortal cell progenitor MDA-MB-231 was more tumorigenic than the SCP type T47D cell line. Female (n = 5 per cell line) and male (n = 4 per cell line) CD1*nude* mice received 5×10^6^ cells by subcutaneous injection and observed up to 47 days for tumor formation. Chart displays mean tumor sizes (± S.D.) generated by MDA-MB-231 and T47D cell lines, respectively in female (top) and mala (bottom) mice. T47D cells formed smaller tumors that regressed and completely resolved within 30 days. Tumors formed by MDA-MB-231 were larger in size and did not show total resolution. Note that this cell line was more tumorigenic in male mice.

Distinct cell-type features associated with SCP and ICP subtypes suggest that they may be phenocopies of molecularly defined breast tumor subtypes [1], [2], [3], [30]. As the prognosis and therapeutic response of each subtype is different [2], [3], we questioned whether we could assign SCP and ICP cell lines to known molecular subtypes of breast tumors. Using cell line and primary tumor gene expression datasets, we conducted a hierarchical clustering analysis. The “intrinsic gene set” data generated by Sorlie et al. [2] to classify breast tumors into five molecular subtypes was used to filter cell line data generated by Charafe-Jauffret et al. [13]. A set of 175 genes was common between the two data sets. Sixty-eight tumors and 31 cell lines were subjected to pair-wise complete-linkage hierarchical clustering and distance measurements. This tumor–cell line combined analysis produced two major clusters. One cluster was composed of basal and luminal B subtype tumors and five of six ICP cell lines. The other cluster included luminal A, ERBB2+, and “normal-like” subtype tumors and all five SCP subtype cells. Four cell lines clustered with the luminal A tumor subclass. Finally. one cell line clustered with the “normal-like” subclass (Fig. 7A). A full list of clustered tumors and cell lines is provided in Fig. S6.

Luminal A tumors clustering with our SCP subtype cell lines displayed the longest tumor-free, distant metastasis-free, and overall survival rates. In contrast, basal and luminal B tumors clustering with our ICP subtype cell lines had the worst prognosis, with shorter tumor-free, distant metastasis-free, and overall survival times [2]. Our cluster analysis suggested that the ability to generate differentiated and senescent progeny characterized breast cancers with poor tumorigenicity, and that resistance to differentiation and senescence was indicative of more aggressive tumorigenicity. We compared in vivo intrinsic tumorigenic behaviors of the SCP cell line T47D and the ICP cell line MDA-MB-231 in female (n = 5 for each cell line) and male (n = 4 for each cell line) CD1 *nude* mice Fig. 7B). MDA-MB-231 cells displayed higher tumorigenicity than T47D cells. In total, we observed progressively growing tumors in five of nine animals injected with MDA-MB-231. In contrast, T47D formed smaller and regressing tumors in nine of nine animals (P<0.03). Interestingly, the difference in tumorigenicity between these two cell lines was more pronounced in male animals (P<0.05 after 20 days post-injection). To test whether the difference in tumorigenicity between MDA-MB-231 and T47D cells was correlated with spontaneous in vivo senescence, we analyzed four tumors from each cell line. Two T47D tumors displayed positive SABG staining, whereas no positive staining was observed with all four MDA-MB-231 tumors (Fig. S7).

## Discussion

In recent years, phenotypic heterogeneity of breast cancers has been correlated with genetic and molecular heterogeneity [1], [3]. Breast cancer subtypes may represent cancers originating from different progenitor cells. Molecular and phenotypic heterogeneity and associated clinical manifestations of breast tumor subtypes have been related to the type of hypothetical tumor progenitor cells originating from a hypothetical mammary epithelial stem cell or from downstream progenitor cells [4], [5]. This hypothesis has not been fully validated, mainly because a hierarchical map of cells involved in mammary epitheliogenesis has not yet been established.

To better understand phenotypic differences between breast cancer subtypes, we applied senescence as a surrogate marker for the potential to generate terminally differentiated progeny. We completed these studies with markers for breast stem/progenitor and differentiated luminal and myoepithelial lineage cells. The use of low-density clonogenic conditions allowed us to follow the fate of a large number of progeny for each cell line studied. From these approaches, we draw several important conclusions. First, breast cancer cell lines form two distinct groups: SCP and ICP subtypes. The SCP cell lines produce non-senescent and senescent progeny, whereas the ICP cell lines produce only non-senescent progeny. Second, SCP and ICP cell lines are exclusively ER+ and ER- cell lines, respectively. Senescence occurs as a result of ER loss associated with p21^Cip1^ induction in SCP cells. Inversely, experimental activation of ER by E2 protects from senescence, whereas its inactivation by tamoxifen aggravates it. Thus, senescence in ER-dependent cells appears to result from the loss of survival signals generated by transcriptional activity of ER. A similar type of senescence has been reported for lymphoma, osteosarcoma, and hepatocellular carcinoma tumors upon c-*MYC* inactivation [31]. Third, SCP cells generate ER+, CD24+, or CK19+ luminal-like, as well as ASMA+ myoepithelial-like progeny. These findings strongly suggest that most, if not all, SCP cells have the capacity to give rise to two major types of differentiated cells that are found in normal mammary epithelium. In sharp contrast, ICP cells never produce ER+ luminal-like or ASMA+ myoepithelial-like cells. Indeed, some ICP cells generate only CD44+ stem/progenitor-like cells and never CD44−, CD24+, CK19+, ER+, or ASMA+ cells. These findings indicate that ICP cells have limited differentiation ability, at least under in vitro conditions. The differentiation ability of ICP cells appears to be lost completely or partially, so that they do not differentiate fully while they self-renew as stem/progenitor-like cells. Fourth, SCP cell lines form the same molecular cluster with luminal A and “normal-like” breast tumors. This suggests that SCP cell lines are phenocopies of these relatively benign and/or anti-estrogen-responsive tumors. The poor tumorigenicity of SCP cells in *nude* mice correlates with better tumor-free and metastasis-free survival of patients with luminal A type tumors. In contrast, ICP cell lines cluster with luminal B and basal-like breast tumor subtypes, and they are more tumorigenic, as expected for luminal B and basal-like tumor cells. It is presently unknown whether breast tumor subtypes that cluster with SCP or ICP cell lines are also composed of either differentiating or mostly self-renewing stem/progenitor cells. Recent studies reported that breast tumors may contain only CD44+, or only CD24+ cells, as well as mixed cell populations, and that CD44+ tumor cells express many stem-cell markers [27], [32]. In addition, an association between basal-like breast cancer and the presence of CD44+/CD24− cells has been established [32].

The mechanisms of the differentiation block observed in ICP cell lines are not known. One might argue that cell lines that produce only CD44+, but never differentiation marker-positive cells, cannot be defined as stem/progenitor cells. However, such cell lines are not completely inert to differentiation stimuli, and may undergo differentiation under special conditions. For example, MDA-MB-231 and MDA-MB-468 cells (identified as ICP cell lines here) can be induced to differentiate into ER+ cells by Wnt5a treatment, and MDA-MB-231 cells then become sensitive to tamoxifen [33].

Most of the findings reported here are derived from in vitro studies performed with established cancer cell lines. Presently, it is unknown to what extent these findings are also relevant for breast tumors. We provide here some promising data that supports in vivo relevance of our conclusions. First, our cluster analyses associated SCP cell types with luminal A and “normal-like” breast tumors, whereas ICP cell types shared similar gene expression profiles with luminal B and basal-like breast tumors. Second, ICP-type MDA-MB-231 cells were more tumorigenic than SCP-type T47D cells. T47D cells formed smaller tumors in some animals, and all tumors displayed regression between 10 and 15 days post-injection. Accordingly, we observed positive SABG staining in two out of four T47D tumors, but not in MDA-MB-231 tumors. Obviously, ICP-like and SCP-like tumors in affected women may or may not display similar tumorigenic potentials, depending on the women's hormonal status and treatment conditions. However, as most SCP-like luminal A or ER+ tumors are successfully treated with tamoxifen [34], their less aggressive behavior could be related to their highly effective senescence response to tamoxifen treatment, as shown here with T47D cells under in vitro conditions (Fig. 3). It will be interesting to examine whether the success of anti-estrogenic treatments is indeed associated with senescence induction in breast tumors. If this is the case, senescence-inducing treatments could be considered for breast cancer.

In conclusion, our analyses reveal that the in vitro ability to generate senescent progeny permits discrimination between cells that share molecular and tumorigenic similarities with luminal A subtype breast tumors from cells related to basal/luminal B subtype tumors. We also provide in vitro evidence for classifying breast cancers into two major groups based on the ability to generate differentiated progeny. Less-tumorigenic SCP cell lines generate both luminal- and myoepithelial-like cells. In contrast, more-tumorigenic ICP cell lines are defective in their ability to generate differentiated progeny. Our findings may have prognostic relevance and serve as a basis for therapeutically inducing differentiation and senescence in breast cancer.

## Supporting Information

Dataset S1 STR analysis data that shows the authenticity of breast cancer cell lines used in this study.
(1.50 MB XLS)

Table S1 Gene clusters, genetic mutations and epigenetic changes of breast cancer cell lines used in this study.
(0.05 MB DOC)

Table S2 Estrogen receptor (ER) status, main pathological features of senescence staining (SABG) of breast tumors used in this study.
(0.05 MB DOC)

Figure S1 p16Ink4a expression in colonies obtained from breast cancer cell lines. There was no correlation between p16Ink4a expression and progenitor subtype.
(3.39 MB TIF)

Figure S2 Co-staining experiments indicate that SABG staining is associated with p21Cip1, but with p16Ink4a expression in SCP cells. Colonies were generated from T47D and MB-MDA-231 cells and subjected to SABG staining, followed by p21Cip1 or p16Ink4a immunoperoxidase (brown) staining. MDA-MB-231 cells were used as negative control.
(2.74 MB TIF)

Figure S3 Effect of estrogen receptor-overexpression on the production of BrdU-negative terminally arrested cell progeny. ER-overexpressing (ER-5, ER-7, ER-26) and control (C-8, C-10, C-11) stable clones were established from T47D cells. Following transfection with ER expression and control vectors, colonies were generated from respective cell lines, labeled with BrdU for 24 h, immunostained for BrdU (brown), and slightly counterstained with hematoxylin to visualize BrdU+ and BrdU- cells.
(6.57 MB TIF)

Figure S4 Detection of SABG+ senescent cells in estrogen receptor-positive breast tumors. Snap-frozen tumors were used to obtain 6 µ thick sections and used directly to detect SABG+ cells. H&E: hematoxylin-eosin staining.
(9.45 MB TIF)

Figure S5 ASMA+ myoepithelial-like cells are produced frequently in senescent cell progenitor T47D and MCF-7 cell lines under confluent conditions. ASMA was tested by immunoperoxidase.
(4.08 MB TIF)

Figure S6 Unsupervised hierarchical clustering of breast tumor and cell line gene expression data that is described in Fig. 7A. Dendrogram shown here includes tumor and cell line sample IDs.
(1.05 MB TIF)

Figure S7 Tumors derived from T47D but not from MDA-MB-231 display SABG (+) senescent cells. Two of four T47D tumors displayed SABG+ cells. All four MDA-MB-231 tumors lacked SABG+ cells.
(7.97 MB TIF)
