# Supplementary material for: The Ability to Generate Senescent Progeny as a Mechanism Underlying Breast Cancer Cell Heterogeneity
Source: PLoS One. 2010 Jun 24;5(6):e11288. doi: 10.1371/journal.pone.0011288 (PMC2891998; doi:10.1371/journal.pone.0011288)
Supplement: Table S1 — Gene clusters, genetic mutations and epigenetic changes of breast cancer cell lines used in this study. (0.05 MB DOC) [file pone.0011288.s002.doc]

**Table S1**: Gene clusters, genetic mutations and epigenetic changes of breast cancer cell lines used in this study

| Cell Line | Gene  Cluster | *PIK3CA* | *CCND1* | *ERBB2* | *KRAS*  *BRAF* | *TP53* | *CDKN2A* | *RB1* | *PTEN* | *NF2* |
| --- | --- | --- | --- | --- | --- | --- | --- | --- | --- | --- |
| T47D | L | M/+ |  |  |  | M/- | met |  |  |  |
| ZR-75-1 | L |  | A |  |  | +/+ | met |  | M/- |  |
| MCF7 | L | M/+ |  |  |  | +/+ | M/- |  |  |  |
| CAMA-1 | L |  | A |  |  | M/- | +/+ |  | M/+ |  |
| BT-474 | L | M/+ | A | A |  | M/- | RNA (-) |  |  |  |
| MDA-MB-453 | L | M/+ |  |  |  | +/+ | +/+ |  | M/? |  |
| SK-BR-3 | L |  |  | A |  | M/- | unmet |  |  |  |
| BT-20 | BaA | M/M |  |  |  |  | M/- |  | M/- |  |
| MDA-MB-468 | BaA |  |  |  |  | M/ - | +/+ | M/- | M/? |  |
| HCC1937 | BaA | +/+ |  |  |  | M/- | +/+ |  | -/- |  |
| MDA-MB-231 | BaB |  |  |  | M/+  M/+ | M/- | M/- |  |  | M/- |
| MDA-MB-157 | BaB | +/+ |  |  |  | M/- | +/+ |  |  |  |
| References | 21 | Sanger^1^ | 21 | 21 | Sanger^1^ | Sanger^1^, 44 | Sanger^1^, 21, 43 | Sanger^1^ | Sanger^1^, 42 | Sanger^1^ |

A, amplified; BaA, basal A; BaB, Basal B; L, luminal; M, mutated; met, promoter methylated; unmet, promoter unmethylated; +/+, wild-type; -/-, homozygous deletion; ?, unknown. ^1^From Sanger COSMIC data (http://www.sanger.ac.uk/genetics/CGP/cosmic/).
