# Supplementary material for: The Ability to Generate Senescent Progeny as a Mechanism Underlying Breast Cancer Cell Heterogeneity
Source: PLoS One. 2010 Jun 24;5(6):e11288. doi: 10.1371/journal.pone.0011288 (PMC2891998; doi:10.1371/journal.pone.0011288)
Supplement: Table S2 — Estrogen receptor (ER) status, main pathological features of senescence staining (SABG) of breast tumors used in this study. (0.05 MB DOC) [file pone.0011288.s003.doc]

**Table S2**: Estrogen receptor (ER) status, main pathological features of senescence staining (SABG) of breast tumors used in this study

| **Sample Label** | **Patient age** | **Menopause** | **ER status** | **Grade** | **LN** | **Stage** | **Type** | **SABG** |
| --- | --- | --- | --- | --- | --- | --- | --- | --- |
| MFT29 | 58 | Post-menopause | (+) | 1 | (+) | 2 | IDC | Negative |
| MFT46 | 64 | Post-menopause | (+) | 1 | (+) | N/A | IDC | Negative |
| MFT55 | 68 | Post-menopause | (+) | 2 | (-) | N/A | IDC | Negative |
| **MFT58** | **62** | **Post-menopause** | **(+)** | **3** | **(+)** | **2B** | **IDC** | **Positive** |
| MFT78 | 69 | Post-menopause | (+) | 2 | (+) | N/A | IDC | Negative |
| MFT83 | 47 | Post-menopause | (+) | 2 | (+) | 2B | IDC | Negative |
| MFT88 | 60 | Post-menopause | (+) | 2 | (+) | N/A | IDC | Negative |
| MFT90 | 32 | Post-menopause | (+) | 2 | (+) | 2B | IDC | Negative |
| MFT93 | 42 | N/A | (+) | 3 | (+) | 2A | IDC | Negative |
| MFT95 | 56 | Post-menopause | (+) | 3 | (+) | N/A | IDC | Negative |
| MFT99 (right) | 68 | Post-menopause | (+) | 2 | (+) | 2A | IDC | Negative |
| **MFT99 (left)** | **68** | **Post-menopause** | **(+)** | **2** | **(+)** | **1** | **IDC+ILC** | **Positive** |
| Mean | 58 |  |  |  |  |  |  |  |
| SD | 12 |  |  |  |  |  |  |  |
|  |  |  |  |  |  |  |  |  |
| N/A, not available. | |  |  |  |  |  |  |  |
