# Supplementary figures and images for: The Ability to Generate Senescent Progeny as a Mechanism Underlying Breast Cancer Cell Heterogeneity
Source: PLoS One. 2010 Jun 24;5(6):e11288. doi: 10.1371/journal.pone.0011288 (PMC2891998; doi:10.1371/journal.pone.0011288)

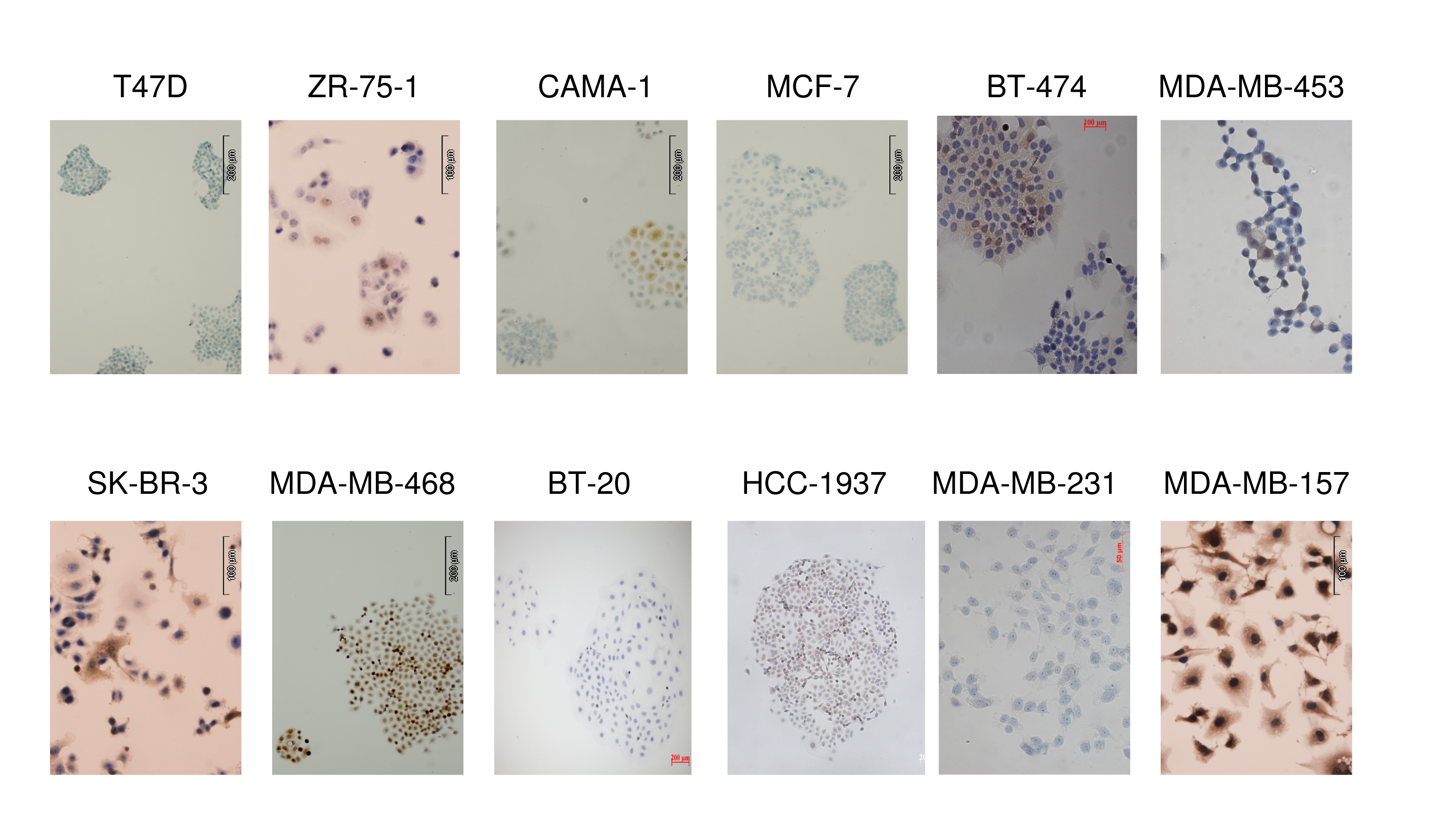

Supplement: Figure S1 — p16Ink4a expression in colonies obtained from breast cancer cell lines. There was no correlation between p16Ink4a expression and progenitor subtype. (3.39 MB TIF) [file pone.0011288.s004.tif]

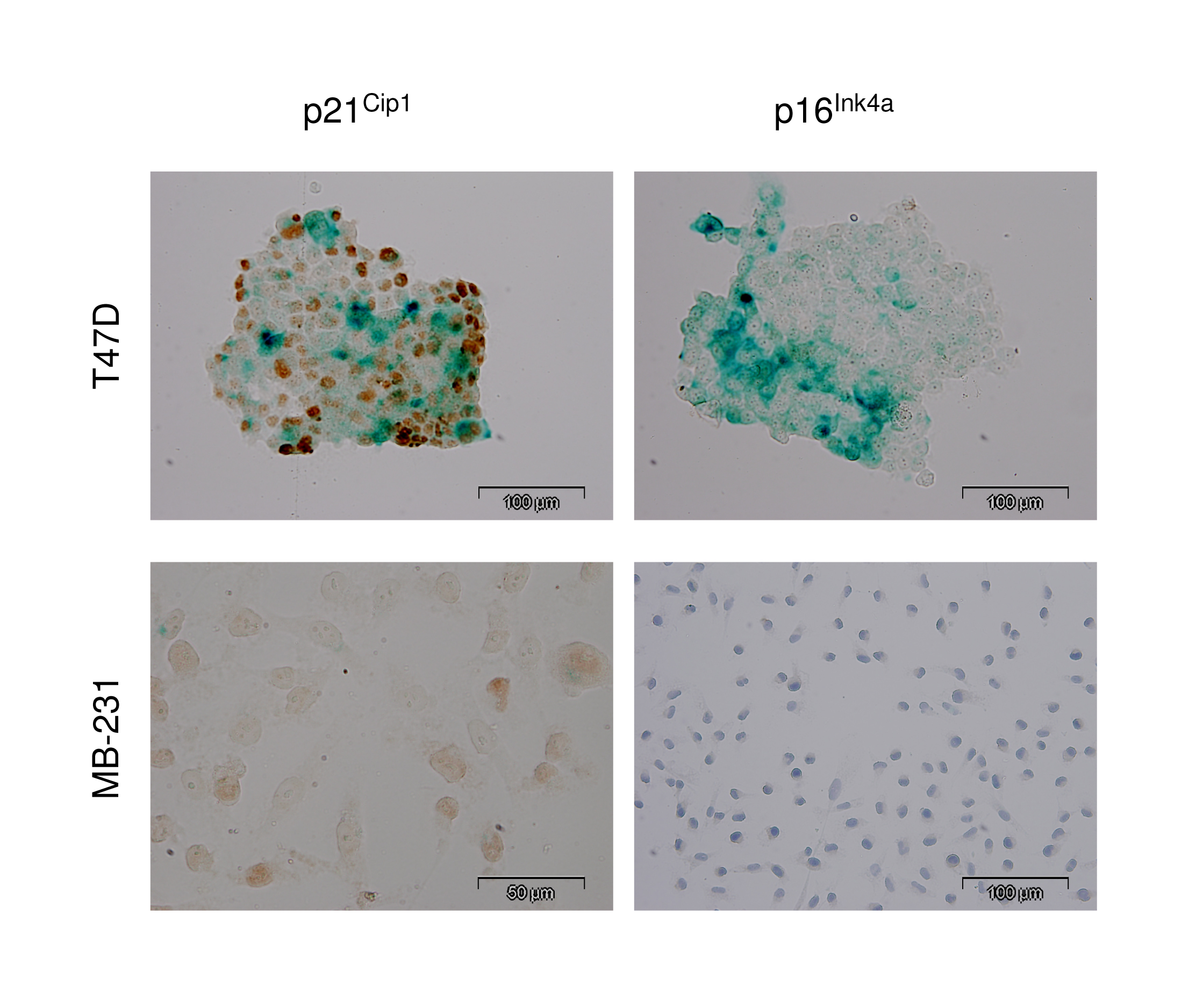

Supplement: Figure S2 — Co-staining experiments indicate that SABG staining is associated with p21Cip1, but with p16Ink4a expression in SCP cells. Colonies were generated from T47D and MB-MDA-231 cells and subjected to SABG staining, followed by p21Cip1 or p16Ink4a immunoperoxidase (brown) staining. MDA-MB-231 cells were used as negative control. (2.74 MB TIF) [file pone.0011288.s005.tif]

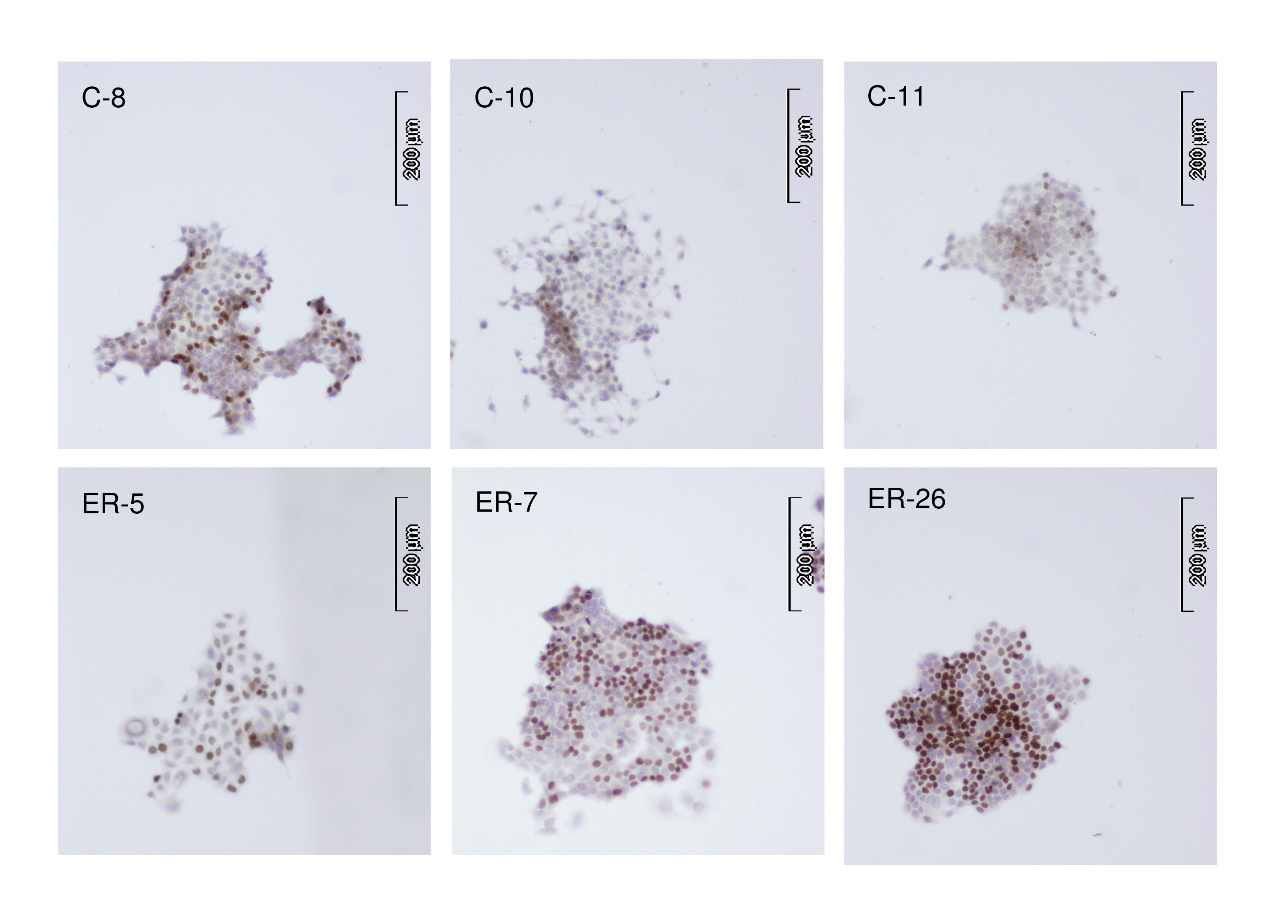

Supplement: Figure S3 — Effect of estrogen receptor-overexpression on the production of BrdU-negative terminally arrested cell progeny. ER-overexpressing (ER-5, ER-7, ER-26) and control (C-8, C-10, C-11) stable clones were established from T47D cells. Following transfection with ER expression and control vectors, colonies were generated from respective cell lines, labeled with BrdU for 24 h, immunostained for BrdU (brown), and slightly counterstained with hematoxylin to visualize BrdU+ and BrdU- cells. (6.57 MB TIF) [file pone.0011288.s006.tif]

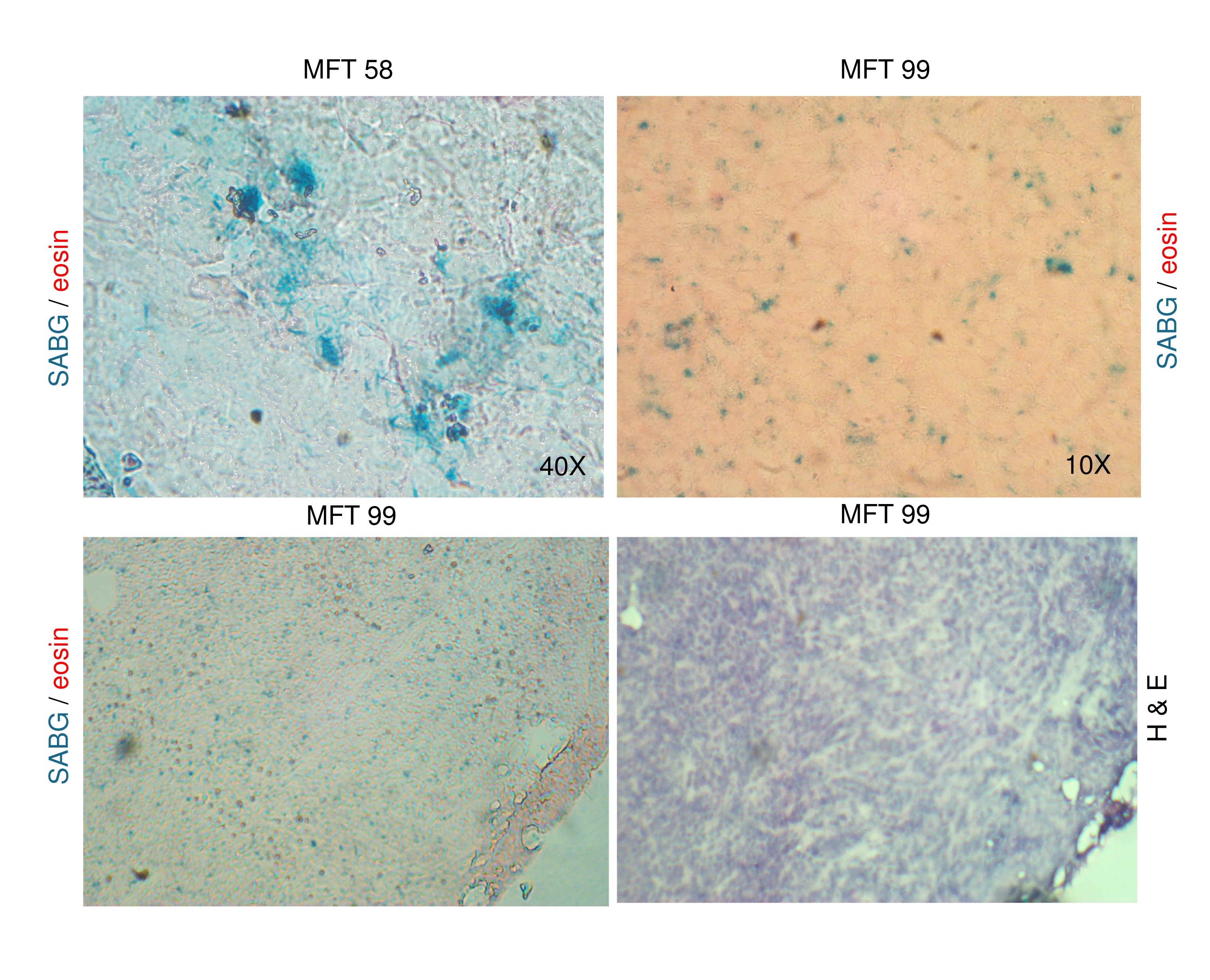

Supplement: Figure S4 — Detection of SABG+ senescent cells in estrogen receptor-positive breast tumors. Snap-frozen tumors were used to obtain 6 µ thick sections and used directly to detect SABG+ cells. H&E: hematoxylin-eosin staining. (9.45 MB TIF) [file pone.0011288.s007.tif]

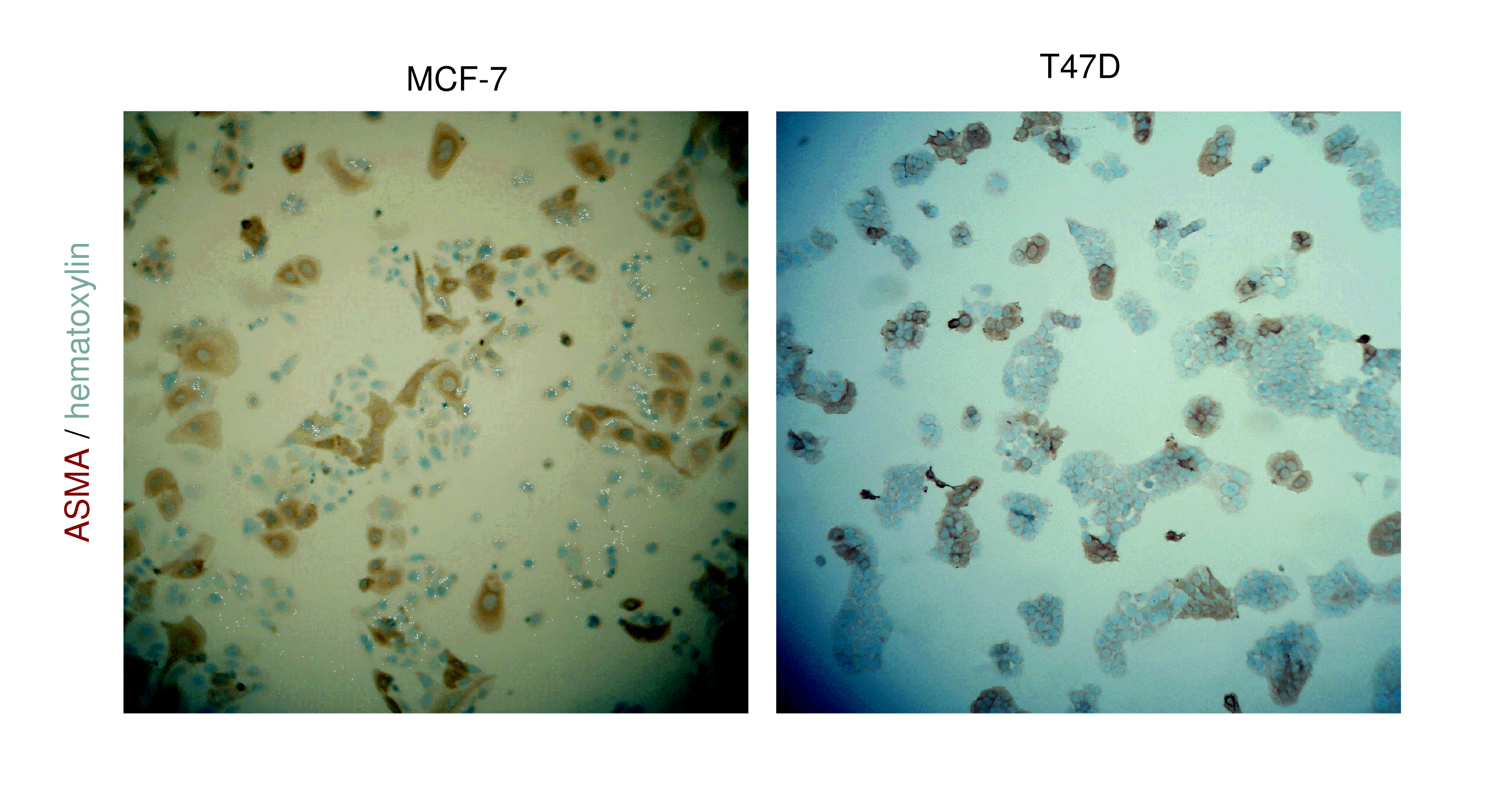

Supplement: Figure S5 — ASMA+ myoepithelial-like cells are produced frequently in senescent cell progenitor T47D and MCF-7 cell lines under confluent conditions. ASMA was tested by immunoperoxidase. (4.08 MB TIF) [file pone.0011288.s008.tif]

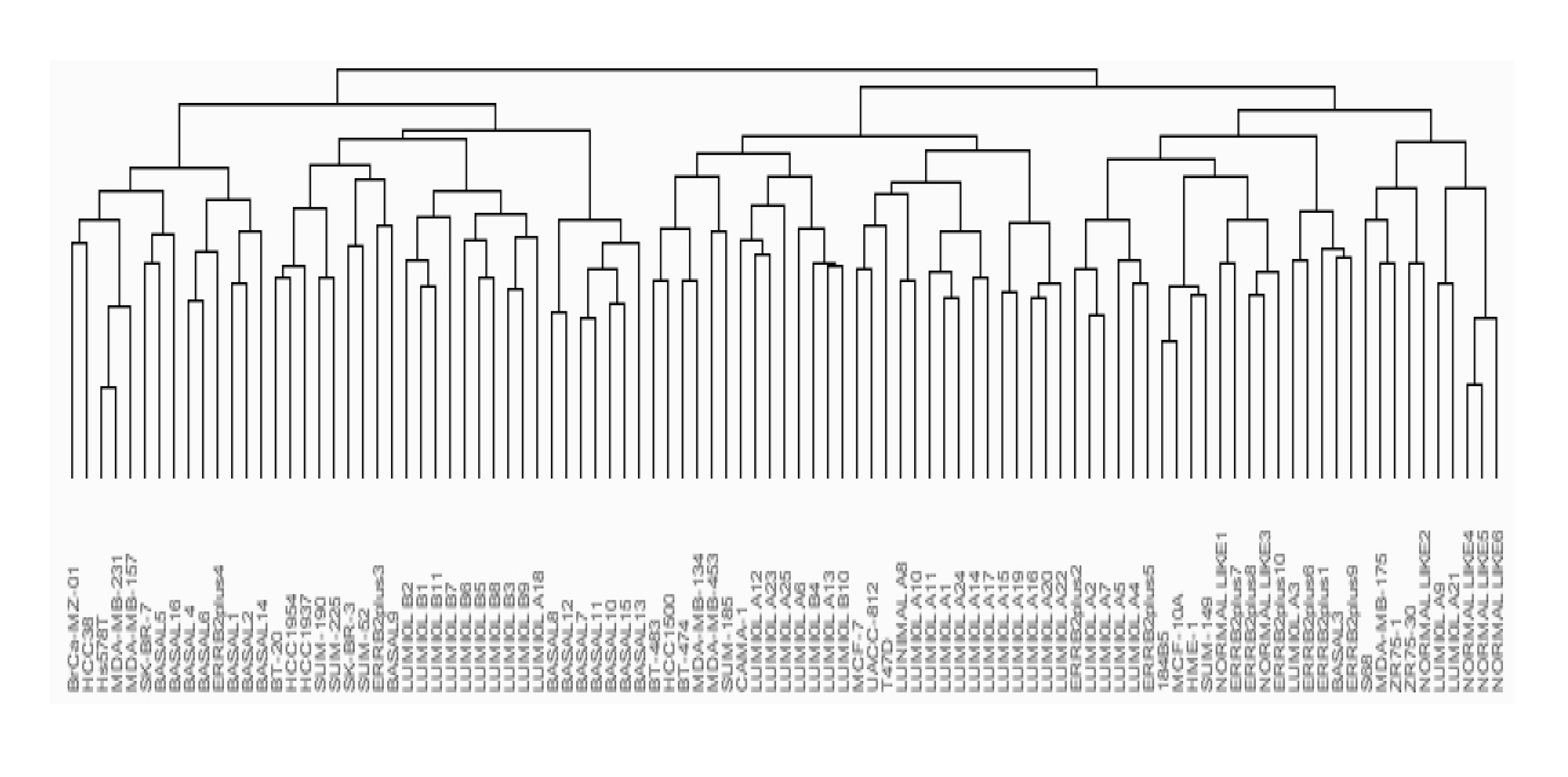

Supplement: Figure S6 — Unsupervised hierarchical clustering of breast tumor and cell line gene expression data that is described in Fig. 7A. Dendrogram shown here includes tumor and cell line sample IDs. (1.05 MB TIF) [file pone.0011288.s009.tif]

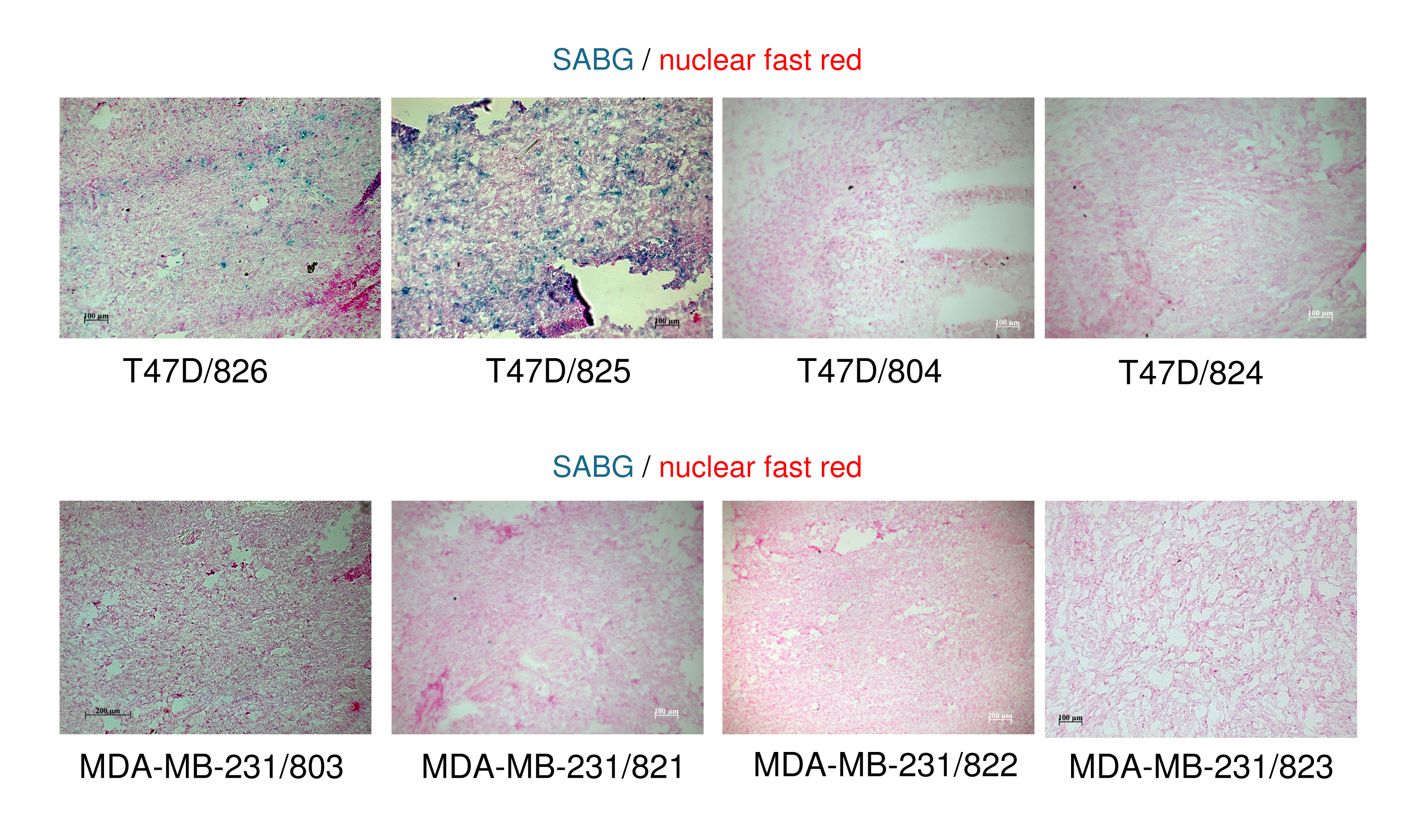

Supplement: Figure S7 — Tumors derived from T47D but not from MDA-MB-231 display SABG (+) senescent cells. Two of four T47D tumors displayed SABG+ cells. All four MDA-MB-231 tumors lacked SABG+ cells. (7.97 MB TIF) [file pone.0011288.s010.tif]
